# A Study on the Prevalence of Laryngopharyngeal Reflux in Saudi Arabia

**DOI:** 10.7759/cureus.59211

**Published:** 2024-04-28

**Authors:** Ali A Ahbail, Hamzah Alhajuj, Tariq Alharbi, Abdulrhman M Alghamdi, Hosam Amoodi, Wed M Salah, Mariam Al Sheikah

**Affiliations:** 1 College of Medicine, University of Jeddah, Jeddah, SAU; 2 Department of Otolaryngology, Head and Neck Surgery, University of Jeddah, Jeddah, SAU; 3 Department of Anatomy, University of Jeddah, Jeddah, SAU

**Keywords:** laryngopharyngeal reflux, reflux symptom index, saudi arabia, gastroesophageal reflux disease (gerd), prevalence, laryngopharyngeal reflux disease

## Abstract

Introduction

Laryngopharyngeal reflux (LPR) is a condition characterized by the backflow of gastric contents rising through the esophagus, affecting the aerodigestive tract and leading to throat symptoms such as hoarseness, chronic cough, and throat clearing. LPR is recognized as a separate condition from gastroesophageal reflux disease, despite the fact that they both involve the backflow of the stomach contents as their primary pathology. Our study aimed to evaluate the prevalence of LPR within the population of Saudi Arabia.

Methods

A cross-sectional study was conducted using an electronic questionnaire from August to November 2023, involving participants from all five regions of Saudi Arabia. A total of 1140 participants completed the questionnaire, which included the Reflux Symptom Index (RSI) to assess the prevalence of LPR.

Results

LPR was found to be prevalent in 31.2% of the study population, with the most common associated demographics being female gender (p = 0.032) and adults aged 36-45 years (p = 0.006). However, no significant relationship was observed based on region of residence or other demographic factors such as education level or occupation.

Conclusion

LPR has a high prevalence in the population of Saudi Arabia. Therefore, further research and awareness about this condition are warranted to better understand its impact, improve diagnosis, and develop appropriate management strategies.

## Introduction

Laryngopharyngeal reflux (LPR) is also known as supra-esophageal reflux, extra-esophageal reflux, or silent reflux. It refers to a disease in which there is a backflow of gastric contents that rises through the esophagus affecting the aerodigestive tract and leading to throat symptoms, specifically those of the laryngopharynx [1]. In some patients, the gastroduodenal content may even reach the nasal cavities and ears through the Eustachian tubes, which can aggravate rhinitis, sinusitis, or otitis media [2-4].

LPR is considered a distinct condition from gastroesophageal reflux disease (GERD), although they share reflux of stomach contents as their main pathology [5]. LPR typically occurs during daytime, while upright, and is not linked to obesity [6]. Patients typically present with symptoms such as post-nasal drip, sore throat, chronic cough, dysphagia, hoarseness, excessive throat mucous, repeated throat clearing, and foreign body sensation within the throat [7]. The worldwide prevalence rate of laryngopharyngeal reflux disease (LPRD) ranges from 5% to 30% [8]. It has been observed that approximately 4-10% of all ENT outpatient clinic consultations are related to manifestations of LPR [9].

In 2022, research conducted on the Indian population revealed that the prevalence rate of LPR in the population was 11%. The prevalence rate of LPR was 11.2% in females and 10.6% in males [8].

In 2021, research conducted in the Chongqing area revealed that the prevalence rate of LPR in ENT clinics was 11.90%. The prevalence rate of LPR was 11.42% in females and 12.55% in males [10].

In 2012, research conducted on the English population revealed that the mean Reflux Symptom Index (RSI) was 8.3. A total of 30% had an RSI higher than 10, and of those, 75% exhibited symptoms of GERD (r = 0.646 at p = 0.01) [7]. Saudi data are not available about the prevalence of LPR. This study mainly aims to discover the same using the RSI score. This will assist in evaluating the burden of LPR disease in Saudi Arabia.

## Materials and methods

Study design, setting, and population

A cross-sectional study was conducted in Saudi Arabia from August to November 2023. The sample size of 385 patients was calculated using an online calculator for cross-sectional studies (Raosoft Sample Size Calculator, Raosoft, Inc., Seattle, WA) [11] based on the total Saudi population of 32,175,224, aiming for a 95% confidence level with a 5% confidence interval. The study targeted both males and females aged 18 to 64 years. The questionnaire was distributed and collected randomly from five regions in Saudi Arabia: the Central Region (Riyadh), Eastern Region (Dammam, Qatif, and Al-Ahsa), Western Region (Makkah, Madinah, and Jeddah), Southern Region, and Northern Region.

Data collection method

An anonymous survey was distributed through Google Forms (Google, Mountain View, CA). The responses were kept anonymous, with no identifying information included. The survey had two sections: the first asked about personal data such as age, gender, region, profession, and marital status, while the second contained questions to evaluate LPR symptoms using the RSI [12].

Reflux symptom index (RSI)

This questionnaire was used to assess LPR symptoms. The validated Arabic version of the RSI was utilized [13]. The questionnaire consisted of nine inquiries, including hoarseness, clearing your throat, excess throat mucus or postnasal drip, difficulty swallowing, coughing after eating or lying down, difficulty breathing, chronic cough, foreign body sensation in your throat, and burning sensation in the mouth or stomach (indigestion or reflux of stomach acid). Each symptom was assigned a score from 0 to 5 based on its severity. The maximum total score was 45. A diagnosis of LPR is suggested if the total score is ≥13, as proposed by Belafsky et al. [12]. The RSI questionnaire was chosen for its established validity and reliability in assessing LPR symptoms.

Statistical analysis

The study employed descriptive statistics to summarize the data, presenting counts, proportions (%), and mean values with standard deviations, as appropriate. To explore the relationship between prevalence and participants' socio-demographic characteristics, statistical analyses were conducted using the Mann-Whitney U test and the Kruskal-Wallis test. A significance level of less than 0.05 (p < 0.05) was considered statistically significant. Additionally, statistical collinearity was assessed using the Shapiro-Wilk test and Kolmogorov-Smirnov test. All statistical analyses were performed using the IBM SPSS version 28 (IBM Corp., Armonk, NY), ensuring robust and reliable results.

Ethical approval

The study was approved by the institutional review board at the University of Jeddah, with application number UJ-REC-165. Prior to participating in the questionnaire, participants provided their consent after receiving a thorough explanation of the study's purpose and procedures.

## Results

Data enrollment

**Figure 1:**
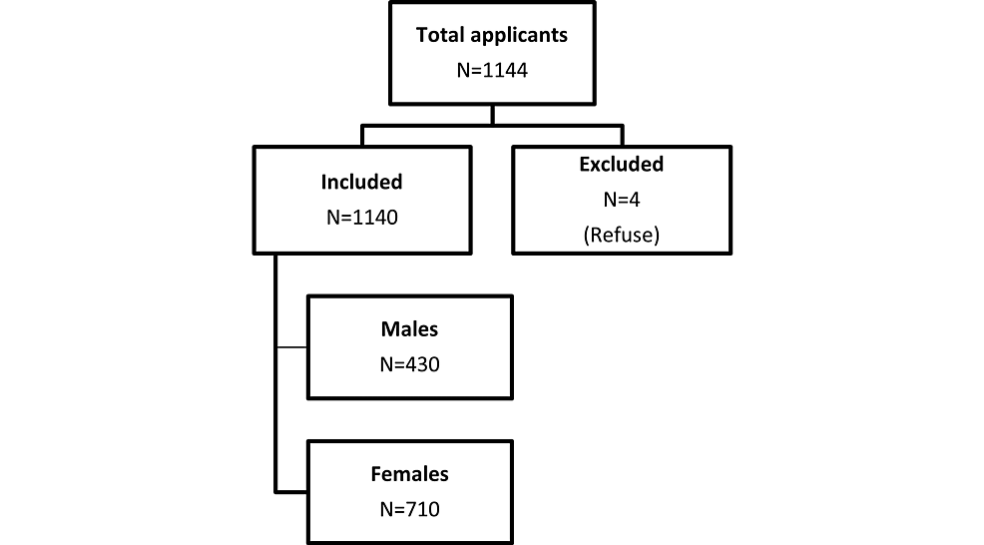
Data enrollment

A total of 1144 individuals applied to participate in the study. Out of these participants, four individuals were excluded, resulting in a final sample size of 1140 participants. The reason for exclusion was individuals chose not to participate and were therefore excluded from the study (Figure 1).

Sociodemographic data

Based on the sociodemographic data, the majority of participants in the study were between 18 and 25 years old (58.3%), with females representing 62.3% of the sample. The vast majority (95.9%) were Saudi nationals, primarily from the eastern (42.7%) and western (28.1%) regions. In terms of marital status, 61.1% of participants were single, while 35.5% were married. Education-wise, the largest group had a bachelor’s degree (51.0%), followed by high school graduates (29.5%). In terms of occupation, 52.8% were unemployed, 32.1% were employed, and the remaining participants were students or retired. Regarding income, 28.0% earned less than 5,000 Saudi riyals (SR), while 18.1% earned more than 20,000 SR (Table 1).

**Table 1:** Sociodemographic data

Sociodemographic data		Count	%
Age	18-25 years	665	58.3%
	26-35 years	164	14.4%
	36-45 years	124	10.9%
	46-55 years	92	8.1%
	More than 55 years	95	8.3%
Gender	Male	430	37.7%
	Female	710	62.3%
Nationality	Saudi	1093	95.9%
	Non-Saudi	47	4.1%
Region	Northern Region	108	9.5%
	Southern Region	72	6.3%
	Eastern Region	487	42.7%
	Western Region	320	28.1%
	Central Region	153	13.4%
Marital status	Single	697	61.1%
	Married	405	35.5%
	Widow	9	0.8%
	Divorced	29	2.5%
Education	Primary	12	1.1%
	Intermediate	23	2.0%
	High school	336	29.5%
	Diploma	129	11.3%
	Bachelor's	581	51.0%
	Master's	43	3.8%
	Ph.D	16	1.4%
Occupation	Unemployed	602	52.8%
	Employed	366	32.1%
	Student	100	8.8%
	Retired	72	6.3%
Income	Less than 5,000 Saudi riyals	319	28.0%
	Between 5,000 and 10,000 Saudi riyals	320	28.1%
	Between 10,000 and 20,000 Saudi riyals	295	25.9%
	More than 20,000 Saudi riyals	206	18.1%

Reflux symptom index

**Figure 2:**
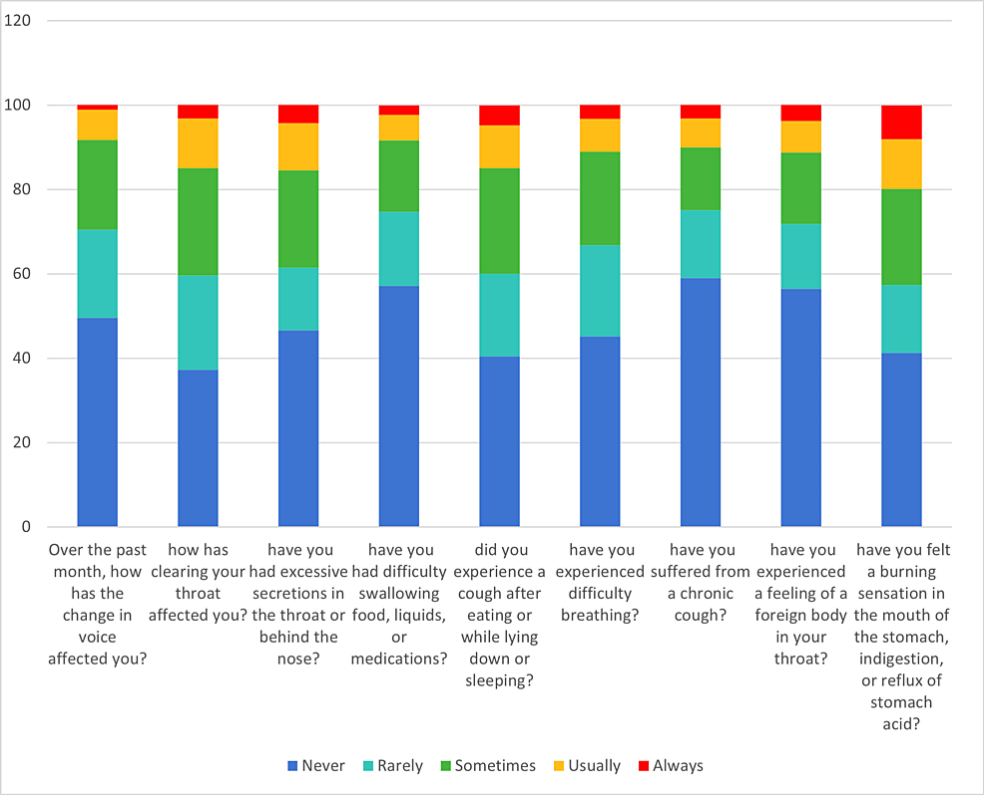
The percentage of Reflux Symptom Index items among study participants

The data indicate symptoms such as a change in voice (7.2% usually, 1.1% always), clearing the throat (11.8% usually, 3.2% always), excessive secretions (11.2% usually, 4.3% always), difficulty swallowing (6.1% usually, 2.2% always), cough after eating or lying down (10.1% usually, 4.7% always), difficulty breathing (7.8% usually, 3.3% always), chronic cough (6.8% usually, 3.2% always), foreign body sensation in the throat (7.5% usually, 3.8% always), and burning sensation in the mouth or stomach (11.8% usually, 8.0% always) (Table 2 and Figure 2).

**Table 2:** The percentage of Reflux Symptom Index items among study participants

	Never, n (%)	Rarely, n (%)	Sometimes, n (%)	Usually, n (%)	Always, n (%)
Over the past month, how has the change in voice affected you?	565 (49.6%)	238 (20.9%)	242 (21.2%)	82 (7.2%)	13 (1.1%)
During the past month, how has clearing your throat affected you?	424 (37.2%)	255 (22.4%)	290 (25.4%)	135 (11.8%)	36 (3.2%)
During the past month, have you had excessive secretions in the throat or behind the nose?	531 (46.6%)	170 (14.9%)	262 (23.0%)	128 (11.2%)	49 (4.3%)
During the past month, have you had difficulty swallowing food, liquids, or medications?	651 (57.1%)	201 (17.6%)	193 (16.9%)	70 (6.1%)	25 (2.2%)
During the past month, did you experience a cough after eating or while lying down or sleeping?	461 (40.4%)	224 (19.6%)	286 (25.1%)	115 (10.1%)	54 (4.7%)
During the past month, have you experienced difficulty breathing?	514 (45.1%)	247 (21.7%)	252 (22.1%)	89 (7.8%)	38 (3.3%)
During the past month, have you suffered from a chronic cough?	673 (59.0%)	183 (16.1%)	170 (14.9%)	78 (6.8%)	36 (3.2%)
During the past month, have you experienced a feeling of a foreign body in your throat?	643 (56.4%)	176 (15.4%)	193 (16.9%)	85 (7.5%)	43 (3.8%)
During the past month, have you felt a burning sensation in the mouth of the stomach, indigestion, or reflux of stomach acid?	470 (41.2%)	185 (16.2%)	259 (22.7%)	135 (11.8%)	91 (8.0%)

Prevalence of laryngopharyngeal reflux in Saudi Arabia

**Figure 3:**
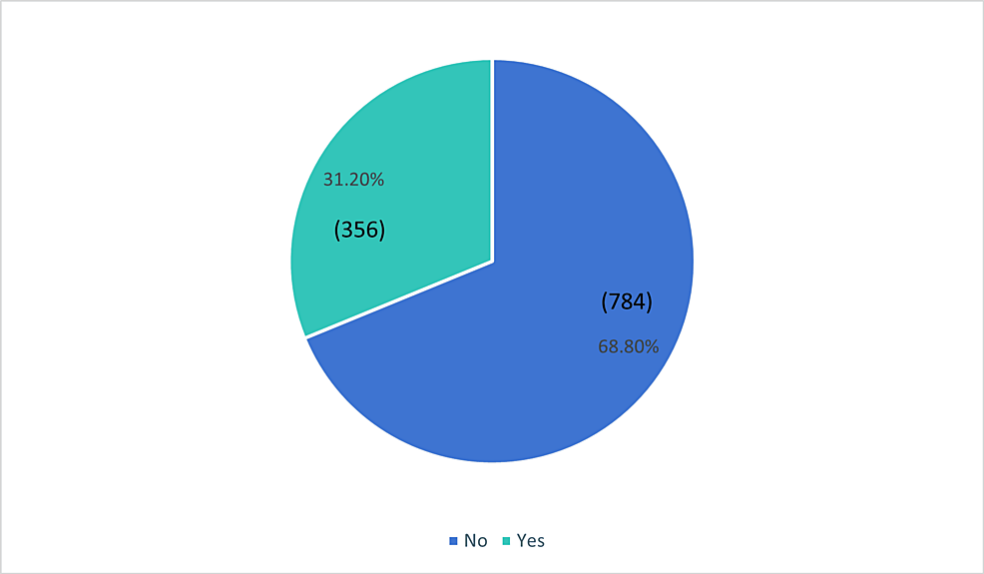
Prevalence of laryngopharyngeal reflux in Saudi Arabia

Based on the data, we found 68.8% of the participants did not had LPR, while 31.2% had LPR (Figure 3).

Assessment of the demographic characteristics of laryngopharyngeal reflux disease patients

Several sociodemographic factors showed a significant association with LPR status. Regarding age, there was a significant association between age groups and LPR status (p = 0.006). The data indicated that individuals in the 36-45 years age group had a higher percentage of LPR (40.3%) compared to the overall population. Furthermore, gender was also found to be significantly associated with LPR status (p = 0.032). The proportion of females with LPR (33.5%) was higher than that of males (27.4%). Marital status showed a significant association with LPR status (p = 0.013). Lastly, income was found to have a significant association with LPR status (p = 0.004). Individuals with an income of less than 5,000 SR had a higher LPR prevalence (35.4%) compared to the overall population (Table 3).

**Table 3:** Demographic characteristics of LPR patients a: Kruskal-Wallis test; b: Mann-Whitney test. LPR: laryngopharyngeal reflux; SR: Saudi riyal.

		LPR status				P-value
		No		Yes		
Sociodemographic data		Count	%	Count	%	
Age^ a^	18-25 years	475	71.4%	190	28.6%	0.006
	26-35 years	118	72.0%	46	28.0%	
	36-45 years	74	59.7%	50	40.3%	
	46-55 years	59	64.1%	33	35.9%	
	More than 55 years	58	61.1%	37	38.9%	
Gender^ b^	Male	312	72.6%	118	27.4%	0.032
	Female	472	66.5%	238	33.5%	
Nationality^ b^	Saudi	752	68.8%	341	31.2%	0.917
	Non-Saudi	32	68.1%	15	31.9%	
Region^ a^	Northern Region	75	69.4%	33	30.6%	0.286
	Southern Region	40	55.6%	32	44.4%	
	Eastern Region	363	74.5%	124	25.5%	
	Western Region	200	62.5%	120	37.5%	
	Central Region	106	69.3%	47	30.7%	
Marital status^ a^	Single	498	71.4%	199	28.6%	0.013
	Married	263	64.9%	142	35.1%	
	Widow	3	33.3%	6	66.7%	
	Divorced	20	69.0%	9	31.0%	
Education^ a^	Primary	5	41.7%	7	58.3%	0.360
	Intermediate	15	65.2%	8	34.8%	
	High school	246	73.2%	90	26.8%	
	Diploma	82	63.6%	47	36.4%	
	Bachelor's	399	68.7%	182	31.3%	
	Master's	26	60.5%	17	39.5%	
	Ph.D	11	68.8%	5	31.3%	
Occupation^ a^	Unemployed	413	68.6%	189	31.4%	0.783
	Employed	248	67.8%	118	32.2%	
	Student	77	77.0%	23	23.0%	
	Retired	46	63.9%	26	36.1%	
Income^ a^	Less than 5,000 SR	206	64.6%	113	35.4%	0.004
	Between 5,000 and 10,000 SR	212	66.3%	108	33.8%	
	Between 10,000 and 20,000 SR	211	71.5%	84	28.5%	
	More than 20,000 SR	155	75.2%	51	24.8%	

The impact of independent variables on RSI: A linear regression analysis

The linear regression analysis revealed several significant associations between the independent variables and the RSI. Among the age categories, individuals aged 36-45 years, 46-55 years, and more than 55 years have significant positive effects on the RSI compared to the reference category of 18-25 years. Specifically, there was an average increase of approximately 2.244 (p = 0.009, 95% CI: 0.572-3.916), 2.255 (p = 0.019, 95% CI: 0.369-4.140), and 4.160 (p < 0.001, 95% CI: 1.836-6.484) units in the RSI, respectively. Additionally, being female compared to male was associated with a statistically significant increase of approximately 1.125 units in the RSI (p = 0.039, 95% CI: 0.057-2.192). In terms of region, individuals from the southern region have a significant positive effect, with an average increase of around 2.701 units (p = 0.019, 95% CI: 0.454-4.948). Lastly, income categories also showed significant associations, with individuals earning less than 5,000 SR having an average increase of about 1.542 units (p = 0.032, 95% CI: 0.131-2.954) compared to the reference category of more than 20,000 SR (Table 4).

**Table 4:** Linear regression analysis Ref. Cat: reference category; SR: Saudi riyal.

	Unstandardized coefficients		Standardized coefficients	t	Sig.	95% CI	
	B	S.E.	Beta			Lower	Upper
Constant	8.668	3.038		2.853	0.004	2.706	14.629
18-25 years (Ref. Cat)							
26-35 years	0.388	0.730	0.018	0.531	0.595	-1.045	1.821
36-45 years	2.244	0.852	0.092	2.634	0.009	0.572	3.916
46-55 years	2.255	0.961	0.081	2.346	0.019	0.369	4.140
More than 55 years	4.160	1.184	0.152	3.512	<0.001	1.836	6.484
Male (Ref. Cat)							
Female	1.125	0.544	0.072	2.067	0.039	0.057	2.192
Northern Region (Ref. Cat)							
Southern Region	2.701	1.145	0.087	2.358	0.019	0.454	4.948
Eastern Region	-0.886	0.844	-0.058	-1.049	0.295	-2.542	.771
Western Region	1.015	0.845	0.060	1.202	0.230	-0.642	2.672
Central Region	0.107	0.956	0.005	0.111	0.911	-1.770	1.983
More than 20,000 SR (Ref. Cat)							
Less than 5,000 SR	1.542	0.720	0.092	2.144	0.032	0.131	2.954
Between 5,000 and 10,000 SR	1.028	0.706	0.061	1.455	0.146	-0.358	2.414
Between 10,000 and 20,000 SR	0.301	0.701	0.017	0.429	0.668	-1.075	1.677

## Discussion

LPR has long been understudied, and its symptoms often indicate other diagnoses of respiratory tract disease. This misjudgment may be attributed to the lack of emphasis regarding this disease in the current literature. The purpose of our study was to assess the prevalence of LPR in the general population of Saudi Arabia. Across the globe, LPR prevalence varies significantly; our study results revealed a 31.2% (356 participants) prevalence rate, meaning that 31.2% of the sample scored ≥13 on the RSI. A different Saudi study introduced by Fahad Z. Alotaibi et al. revealed yet a considerably higher prevalence rate of 51.2% (85 participants) [14]. On the lower end of the LPR prevalence spectrum, Willybroad A. Massawe et al. reported a prevalence rate of 18.4% of their sample [15]. A study conducted over 2300 individuals in India revealed an LPR prevalence rate of 11% (253 participants) [8]. This discrepancy in prevalence rate should prompt the development and introduction of more sophisticated means of diagnosis.

Age and gender were notable demographic variables that illustrated varying results when contrasted against Fahad Z. Alotaibi et al.' paper, a recent Saudi article published in this regard. More than half of our sample fell in the 18-25 years old age group, and only about 10% were 36-45 years of age. Interestingly, our study results have indicated the latter group had a higher prevalence rate of LPR compared to the younger group. This pattern, however, was unobserved in Fahad et al.'s paper, and their results showed a higher prevalence of LPR, accounting for 36.5% amongst their 21-35 years age group and only about 29.4% amongst their 36-50 years age group [14]. Moreover, their results established lower odds of increased LPR risk in their younger group compared to the 1.3 times increased odds of LPR risk in the older group [14]. We think this observation of dissimilarity ought to encourage the following: (1) closely observing and recognizing age in upcoming research while providing a sufficient sample from each age group; (2) following a standardized age grouping system for LPR data to avoid overlapping results; (3) conducting analytic studies utilizing different tools of analysis to associate/disassociate age as a risk factor of LPR. Gender, on the other hand, proceeded in the same direction across the two studies, where we established LPR to be more prevalent amongst females, and Fahad et al. stated that males had lower odds of an increased LPR risk when compared to females [14]. At the current state of literature, we are unable to draw conclusions on either age or gender. However, our observations can stimulate and or formulate new hypotheses concerning LPR.

Limitations

Our study focused on two objectives: (1) the prevalence of LPR amongst the Saudi population and (2) the occurrence rates of demographic and symptomatologic variables in LPR. Consequently, we have stumbled upon a globally dissimilar disease distribution with no rational reasoning behind such rates. This may be the case with most topics with scarce analytical data. We have also come across an apparent shortage of demographic data. Therefore, we attempted to collect as much demographic data as possible without altering sampling techniques or risking bias while merely relying on an extended duration of data collection. Unfortunately, and in a similar fashion to current literature, our study also suffers from a disproportionate age group sample, where most of our sample (58.3%, 655 participants) belong to one age group, namely, 18-25 years of age. While practically valid, this decreases the potential of future hypotheses to be generated, knowing that other groups may need to be sufficiently emphasized. Gender has also been approached similarly, yet females still overtake their male counterparts with a 62.3% occurrence rate (710 participants) of the whole sample. Our study was limited by the absence of a precise validation of LPR diagnosis, such as laryngoscopy and pH monitoring, which would have offered more concrete evidence for our findings. However, the findings from our study can still provide valuable insights and generate hypotheses for further research on LPR demographics and symptomatology.

## Conclusions

LPR's burden has shown to be evident across the globe. The current inconsistency in the disease's distribution, paucity in risk-outcome results, and vague symptomatology necessitate further assessment through upcoming endeavors. Research designs with causation and or risk-determining properties can be implemented on various elements in this topic. Such endeavors are encouraged and are expected to yield a satisfactory amount of information. All in all, we aspire to alleviate this disease’s burden through adequate research to guide one's diagnostic capability and consequently provide accurate treatment options.
